# Features of hemodynamic and immunological parameters in patients with recurrent uveitis complicated by hypertension, Fuchs heterochromic uveitis and Posner-Schlossman syndrome

**DOI:** 10.22336/rjo.2023.5

**Published:** 2023

**Authors:** Natalia Ivanivna Khramenko, Liudmyla Mikolaivna Velychko, Natalia Valerievna Konovalova, Oleksandra Viktorivna Bogdanova, Lilia Dumbrăveanu Gheorghe, Doina Vasile Bobescu

**Affiliations:** * SI “The Filatov Institute of Eye Diseases and Tissue Therapy of the National Academy of Medical Sciences of Ukraine”, Odesa, Ukraine; ** Department of Ophthalmology and Optometry, “Nicolae Testemiţanu” State University of Medicine and Pharmacy, Republic of Moldova, Chişinău

**Keywords:** idiopathic anterior uveitis, Fuchs syndrome, Posner-Schlossman syndrome, T-cell sensitivity to eye antigens, eye hemodynamics

## Abstract

**Introduction:** Uveitis is a disease that manifests with increased vascular permeability and occlusion, with some ischemia and inflammatory mediators. It is characterized by a wide range of pathological processes, including inflammation, increased vascular permeability and occlusion, local ischemia and cell alteration by inflammatory mediators, and is characterized by the presence of complications.

**Aim:** To study the state of ocular hemodynamics by rheoophthalmography, as well as the immune status in patients with idiopathic recurrent anterior uveitis complicated by intraocular hypertension, Fuchs heterochromic uveitis, Posner-Schlossman syndrome, during the relapse period.

**Materials and methods:** 93 patients with idiopathic recurrent anterior uveitis were included in this study, 8 patients with Fuchs’ uveitis, and 6 patients with Posner-Schlossman syndrome. According to clinical signs, relapse and remission were considered. The control group (healthy volunteers of the same age) consisted of 27 people. In this regard, 5 groups of subjects were formed. The mean age of the patients was (M ± SD) 39.2 ± 14.6 years. According to the Median (range), the duration of the disease in patients was 2033 (350-3285) days, intraocular hypertension being recorded at P0 > 20 mm Hg.

*Statistical analysis* was carried out in spreadsheets using STATISTICA 8.0 (StatSoft.Inc) program. Quantitative indicators were evaluated according to the correspondence to the normal distribution and to the Kolmogorov-Smirnov criterion. With a normal distribution, arithmetic means (M) and standard deviations (SD), limits of the 95% confidence interval (95% CI) and Student’s t-test were calculated.

**Results:** The volumetric blood filling of the eye according to the rheoophthalmographic indicator RQ during the period of remission of uncomplicated and complicated by hypertension anterior uveitis was reduced by 32.4%-40.5%, respectively, compared with the norm. During the period of relapse, RQ was significantly higher by 28% (p<0.05) than in remission, in the group of uncomplicated uveitis, and in the group of uveitis with increased IOP, no significant differences between the periods of remission and relapse were observed, which reflected the ischemic process in the relapse period. Volumetric blood filling in Fuchs and Posner-Schlossman syndromes in the acute period did not differ from the norm. Cellular immunity in the groups of uncomplicated and complicated by intraocular hypertension idiopathic uveitis, as well as with Fuchs and Posner-Schlossman syndromes, had a higher level of CD4 helper lymphocytes and a lower level of CD8 suppressor lymphocytes, which reflected higher values of the immunoregulatory index. The increase in the immunoregulatory index is most pronounced in Fuchs and Posner-Schlossman syndromes.

**Discussion:** In the presented study, the incidence of idiopathic recurrent anterior uveitis complicated by intraocular hypertension was 9,9% among all cases of idiopathic recurrent anterior uveitis in one-time period. According to literature, this complicated form of uveitis occurs in 11,5%-46,1% of cases. Most often (up to 92% of cases), the anterior chamber angle was open.

**Conclusions:** Different activity of the mechanisms regulating the balance of cellular and humoral immunity, sensitivity of T-cells to eye antigens in idiopathic anterior uveitis, Fuchs and Posner-Schlossman syndromes was assumed. Peculiarities of eye hemodynamics in these forms of uveitis were also revealed.

**Abbreviations:** IOP = intraocular pressure, IOHS = inflammatory ocular hypertension syndrome, HSV = herpes simplex virus, CMV = cytomegalovirus, OCT = optical coherence tomography, OD = right eye, OS = left eye

## Introduction

Uveitis is one of the most common eye diseases, has many clinical manifestations with different phenotypes, is the fifth leading cause of visual impairment in developed countries, and accounts for about 10% of reported cases of blindness [**1**]. Recurrent anterior uveitis of unknown etiology (or idiopathic uveitis) is the most common form of uveitis [**2**,**3**]. This nosological form is registered in 36% of cases [**4**]. One of the most serious vision-threatening consequences of this condition is secondary glaucoma, which has an incidence of 5% to 24%. Therefore, in uveitis, monitoring of IOP is necessary at all visits for the patient [**5**]. Both acute and transient increases in intraocular pressure are observed against the background of acute or chronic recurrent inflammation [**6**,**7**].

The etiology of uveitis complicated by elevated IOP is different. Intraocular hypertension occurs in both non-infectious (about 15% of cases) [**5**] and infectious uveitis. 25% of uveitic patients have elevated IOP and 20% develop inflammatory glaucoma [**8**]. An increase in IOP is described in viral uveitis: the most common viruses in this case are herpes simplex (HSV), chickenpox, cytomegalovirus (CMV) and rubella. Currently, the following syndromes are associated with viral etiology: Posner-Schlossman syndrome and Fuchs uveitis syndrome. In Posner-Schlossman syndrome, CMV appears to be the main cause, especially in Asia, and less commonly, herpes simplex virus (HSV). As for Fuchs uveitis syndrome, rubella virus is the main etiological agent in the USA and Europe, while CMV is the predominant cause in Asia [**9**].

Posner-Schlossman syndrome is a type of uveitis that is characterized by an acute unilateral increase in IOP, manifested as a glaucomacyclitis crisis, usually of a recurrent nature of the course, characterized by the presence of inflammatory cells in the anterior chamber, rare precipitates, atrophy of the iris, while posterior synechiae are usually absent. Fuchs uveitis (FU) is characterized by chronic, mild anterior uveitis with stellate precipitates, heterochromia and iris atrophy may be present, and early cataracts are common. Patients with FU are characterized by prolonged, mild inflammation and a less acute increase in IOP than with Posner-Schlossman syndrome. This long-term inflammation and persistently elevated IOP are more likely to lead to secondary glaucoma in patients with Fuchs uveitis [**10**]. More than half of HSV-induced keratouveitis develops intraocular hypertension and secondary open-angle glaucoma during relapses, and most of them resolve after proper inflammation treatment [**11**]. Less common causes include sarcoidosis, toxoplasmosis, listeriosis, and syphilis [**6**]. Also, in literature, the etiology of ocular hypertension is considered according to the type of inflammation. Concordant with some data, 76.7% of intraocular hypertension in cases of non-granulomatous uveitis was caused by steroids, but 70% of intraocular hypertension in granulomatous uveitis was induced by inflammation [**12**].

The mechanisms of acute increase in IOP in patients with uveitis are numerous, and may include structural changes such as blockage of the pupil, iris bombe, causing closure of the angle of the anterior chamber, the formation of peripheral anterior synechia, limiting outflow, displacement of the iris root anteriorly against the background of exudative detachment of the ciliary body, and direct inflammation of the trabecular meshwork, which can lead to the phenomenon previously described as inflammatory ocular hypertension syndrome (IOHS) [**13**]. It should be noted that damage to the function of the trabecular meshwork because of a chronic inflammatory process occurs in most cases of intraocular hypertension, and this process is an open-angle type [**14**-**16**].

Ocular hypertension in uveitis is usually associated with resistance to the outflow of aqueous humor associated with exposure to the trabecular meshwork of inflammatory cells, cytokines, iris pigment, corticosteroids, or possibly other factors [**17**]. Ocular hypertension can lead to glaucoma with damage to the optic nerve; therefore, understanding the risk of its occurrence, determined by its predictors, is of great clinical importance [**18**]. Ocular hypertension is considered the most important risk factor for glaucoma; lowering IOP is the main strategy for preventing glaucoma in patients at risk and slowing its progression in patients with advanced glaucoma. According to the results of a retrospective, multicentered, cohort study, statistically significant risk factors for ocular hypertension in uveitis included the presence of systemic hypertension, surgery on the affected or fellow eye, including vitrectomy; presence of structural changes in the anterior (peripheral synechiae) and posterior segment of the eye (epiretinal membrane) active inflammatory process (cells of the anterior chamber: Tyndall +++), low visual function, current use of systemic, periocular and local corticosteroids for the last three months [**5**,**14**]. The treatment of uveitic (inflammatory) glaucoma is particularly challenging because it aims to reduce both inflammation and lower intraocular pressure, the goals of which may conflict with each other [**5**].

The presence of vascular dysregulation in glaucoma is a well-known fact [**19**-**21**]. Depending on the periods of the inflammatory process, features of eye hemodynamics in anterior uveitis are described in some works [**22**,**23**]. We indicated above that systemic hypertension is one of the risk factors, but there is practically no information about the features of regional hemodynamics in anterior uveitis complicated by intraocular hypertension. It is known that the level of volumetric blood filling, perfusion pressure and the vascular wall tonus determine the degree of the pathological process in tissue nutrition.

Experts note that we know little about the immune mechanisms involved in uveitis and, particularly, in idiopathic form [**4**,**23**]. Considering the complex pathogenetic mechanism of uveitis, it is important to study the interaction of the immune and vascular systems, as well as prevention of irreversible morphological complications.

## Aim

To study the state of ocular hemodynamics using rheoophthalmography, as well as the immune status in eyes with idiopathic recurrent iritis complicated by intraocular hypertension, Fuchs heterochromic uveitis, and Posner-Schlossman syndrome during the relapse period.

## Materials and methods

The examination was completed at the Inflammatory Eye Pathology Department, Functional Research Methods Laboratory, Immunology Laboratory of “Filatov Institute of Eye Diseases and Tissue Therapy of the National Academy of Medical Sciences of Ukraine”, in 93 patients with recurrent idiopathic anterior uveitis, 8 patients with Fuchs heterochromic uveitis (Fuchs’ uveitis) and 6 patients with Posner-Schlossman syndrome. According to clinical signs and considering the period of the inflammatory process, we distinguished: relapse and remission, and the presence of complications - intraocular hypertension. The control group (healthy volunteers of the same age) consisted of 27 people. So, 5 groups of subjects were formed. The mean age of the patients was (M ± SD) 39.2 ± 14.6 years. The duration of the disease in patients with idiopathic anterior uveitis, Fuchs heterochromic uveitis, and Posner Schlossman syndrome was similar and was on average 2033 (350-3285) days according to the Median (range). It should be noted that patients with Fuchs and Posner Schlossman syndromes were examined only during the period of relapse due to the lack of observational compliance in the inter-relapse period. All measures to ensure the safety and health, respect for rights, human dignity and moral standards were provided for all patients in accordance with the principles of the Helsinki Declaration of Human Rights, the Council of Europe Convention on Human Rights and Biomedicine and the relevant laws of Ukraine. Written informed consent was obtained from each participant after a detailed explanation of the nature of the study. The institution’s ethics committee approved the work.

All patients underwent clinical ophthalmological examinations, including visual acuity, slit lamp biomicroscopy, gonioscopy, anterior chamber depth measurement, axial length measurement, pachymetry, visual fields (automatic Humphrey examination, standard 24-2 SITA; Carl Zeiss Meditec) and ophthalmoscopy with dilated pupil, OCT of the macula, optic disc and peripapillary retina. Measurement of intraocular hydrodynamics was carried out by tomography; intraocular hypertension was recorded at Р0 > 20 mmHg.

*Rheoophthalmography* (ROG) was performed on a computerized rheographic complex (Reocom, Ukraine, Kharkov). The complex is designed to measure the electrical impedance of human body parts and temporal parameters of electrocardiography (ECG) and process the data obtained, calculate regional hemodynamic parameters, as well as print out the results of measurements and calculations.

Rheoophthalmography (from Greek rheos - flow + ophthalmos - eye + grapho - write) is a method for studying hemodynamics in the choroid, based on continuous graphic registration of changes in the electrical resistance of the eyeball during cardiac cycles. The biophysical foundations of rheography are based on the fact that living tissue is a conductor of electric current, but also has resistance to current. The use of alternating current of high frequency (40-100 kHz) and low power - up to 3 mA made it possible to remove interference and highlight part of the impedance due to fluctuations in the blood filling of tissues. In 1998, the staff of the laboratory and employees of the Kharkov Aerospace University (Ukraine) developed, for the first time in Ukraine, a software for recording and analyzing rheoophthalmography for serial computer rheographs, and developed standard indicators. The presence of anastomoses between the posterior short and long ciliary arteries of the eye allows rheographic examination of the hemodynamics not only in the ciliary body, but also integrally throughout the entire vascular tract and retina.

The study of ROG was carried out with the patient in supine position. Under epibulbar anesthesia with proxymetacaine hydrochloride (0,5%), a contact sensor-lens was placed on the patient’s eyeball and a short-term (up to 60 s) recording was made with parallel ECG recording (lead II).

In this work, we used the parameters of volumetric pulse blood filling, determined by the rheographic coefficient according to Jantsch - (RQ, ‰).

RQ = (A/ Z) * 1000%;

where A is the amplitude of the rheoophthalmogram (Ohm) and Z is the base resistance (Ohm) between the sensor electrodes.

*Immunological studies* included methods for assessing cellular and humoral immunity. Samples were taken by phlebotomy with a disposable syringe, 4-5 ml of heparinized blood being diluted 2 times with 0,9% NaCl solution. Microscopy was performed with a magnification of the objective x 80, eyepiece x 15.

Using the immunohistocytochemical PAP method, using monoclonal antibodies (MAB), the main immunological cells (CD 3 - T cells, CD 4 - T-cells-helper-effectors, CD 8 - T-lymphocytes-suppressors, CD 16 - lymphocytes-killers, CD 19 - B cells) were studied. The levels of immunoglobulins of classes A, M, G were determined by the standard method of enzyme immunoassay using a Statfax apparatus (Awareness Technology, USA), a test system manufactured by Granum, Ukraine was used.

The assessment of the specific reactivity of lymphocytes to eye tissue antigens was carried out using a comprehensive method for assessing the individual sensitivity of the organism to various drugs developed in the laboratory of immunology from Odessa, using the method of parallel trials [**24**]. The technique consists in obtaining a suspension of lymphocytes, and then carrying out their special cultivation with the studied preparations, and the subsequent application of the immunohistochemical PAP method using monoclonal antibodies. The main steps of this technique are as follows: 

- Obtaining a lymphocytic suspension by centrifugation (Ficoll-Verografin solution with a density of 1.076-1.078 g/ cm3) and double cleansing of the cells with balanced salt solution of NaCl 0,9% (BSS).

- Parallel incubation for 1 hour at 370C in an immunological plate: 1) 0,05 ml of cell suspension of lymphocytes and 0,05 ml of BSS; 2) 0,05 ml of a cell suspension of lymphocytes and 0,05 ml of a solution prepared from the retinal tissues; 3) 0,05 ml of a cell suspension of lymphocytes and 0,05 ml of a solution prepared from the tissues of the choroid, 4) 0,05 ml of a cell suspension of lymphocytes and 0,05 ml of a solution prepared from the lens. Obtaining preparations from eye tissues, the concentration of preparation solutions used and the amount of protein in them were developed by previous researchers [**25**]. Measurement of the amount of protein in eye tissue preparations was carried out using a Specol-1300 spectrophotometer, using the Lowry colorimetric method. All preparations had the same amount of protein. Next, a standard immunohistochemical method for determining T-lymphocytes CD 3, using monoclonal antibodies, was used. When considering the results, the number of CD 3 in the experimental samples (with the preparation of the retina, of the choroid and the lens) and the number of CD 3 in the control samples were determined (with BSS) [**26**]. The difference between these parameters was determined, and the obtained data were statistically processed.

*Statistical analysis*

Accumulation, correction, visualization and systematization of the obtained results, and statistical analysis were carried out in spreadsheets using the program STATISTICA 8.0 (StatSoft.Inc). Nominal data were described with absolute values and percentages. Quantitative indicators were evaluated according to the correspondence to the normal distribution according to the Kolmogorov-Smirnov criterion. The data obtained with a normal distribution were combined into variational series. When comparing the average values of normally distributed populations, Student’s t-test was calculated. Sets of quantitative indicators, the distribution of which differed from normal, were described using the values of the median (Median) and the lower and upper quartiles (Ql-Qu). For their comparison, the Mann-Whitney U-test was used. Differences in indicators were considered statistically significant at a significance level of p ≤ 0.05. The strength of correlations was studied by the Spearman and Pearson coefficients.

## Results

Among the cohort of examined patients with idiopathic recurrent anterior uveitis (there were 171 people in the database from 2014 to 2022), a complicated course with an increase in IOP was registered in 17 people (9,9%) and an uncomplicated course was recorded in 76 people (44,4%).

In a complicated course of uveitis with an increase in IOP, a mixed injection of conjunctival vessels, sweating of the corneal endothelium, slight corneal edema, fuzzy iris pattern, and intense opacities in the vitreous body were observed. The optic disc is pale pink, the contours are clear, and the reflexes in the macular zone are fuzzy. The anterior chamber angle (ACA) was open, had an average width, about 30°, the iris root having slight destructive changes.

When studying the relationship of volumetric blood filling of the eye in terms of RQ and the period of the disease (according to the remission code - 0, relapse -1), a direct correlation of weak strength r=0.25 (p<0,05) was revealed. During remission of anterior uncomplicated and complicated by intraocular hypertension uveitis, its decrease in comparison with the norm by 32,4%-40,5% (p<0,05) was noted. During the period of relapse, this indicator does not differ from the norm (**Table 1**). In the uncomplicated course during the period of relapse, RQ was significantly higher by 28% (p<0,05) than in remission, and in the group of uveitis with increased IOP, no significant differences were observed between the periods of remission and relapse. Attention was drawn to the lower values of the upper quartile of the blood filling index in the group of complicated course of uveitis - the tendency to decrease in blood filling in uveitis with intraocular hypertension in all periods of the course of the disease. RQ did not differ from the norm and from the other groups during the relapse period (**Table 1**) in patients with Fuchs and Posner-Schlossman syndromes during the relapse period.

**Table 1:** Volumetric blood filling of the eye RQ (‰) according to rheoophthalmogram data in patients with recurrent anterior uveitis, Fuchs and Posner-Schlossman syndrome

Nosological form	Group number	RQ Volumetric blood filling of the eye	
		Remission	Relapse
		Median	Median
		Range	Range
		n	n
Uncomplicated idiopathic anterior uveitis	1	2,5	3,2
		1,9-3,7	2,1-4,6
		29	47
		Р=0,05	
Complicated by intraocular hypertension idiopathic anterior uveitis	2	2,2	3,1
		1,5-2,6	2,3-3,8
		7	10
		Р=0,1	
Fuchs heterochromic uveitis	3	-	3,8
			3,2-5,2
			8
Posner Schlossman syndrome	4	-	3,6
			3,2-3,8
			6
Control	5	3,7	
		2,2-4,8	
		27	
Significance level of differences		Р1-5= 0,01	Р1-5= 0,3
		Р2-5 = 0,04	Р2-5 = 0,2
			Р3-5= 0,6
			Р4-5= 0,8
**Note:** Median = median; Ql-Qup-lower = upper quartile (25%-75%); M = arithmetic mean; SD = standard deviation; N = number of patients			

When considering the characteristics of cellular immunity in groups of patients with uveitis, it was found that the absolute number of CD3 lymphocytes in groups 1-4 was higher than normal by an average of 20% (p<0,05), but did not differ between groups. The absolute number of CD4 helper lymphocytes was higher than normal in the 1st and 3rd groups, and the relative number of CD4 cells was higher in groups 3-4 by 21% (p<0,05) compared to the norm (**Table 2**). In Posner-Schlossman syndrome, the level of CD4 was higher than in idiopathic uveitis. In the Posner-Schlossman syndrome group, the absolute number of CD8 suppressor lymphocytes was less than normal and less than in Fuchs uveitis (p<0,05). The immunoregulatory index, which reflects the ratio of the number of helper lymphocytes to the number of suppressor cells, was higher than normal in groups 1-4 (**Table 2**) and in the group with Posner-Schlossman syndrome and Fuchs uveitis was higher than in uncomplicated idiopathic uveitis. Thus, a feature of cellular immunity in groups with complicated uveitis and syndromes was a higher level of CD4 helper lymphocytes and a lower level of CD8 suppressor lymphocytes, which reflected higher values of the immunoregulatory index.

**Table 2:** Indicators of cellular immunity during relapse period

Nosological form	Group number	СD 3		СD4		СD8		СD4/ СD8 (immuno-regulatory index)
		cells/ μL	%	cells/ μL	%	cells/ μL	%	%
		Median	M ± SD	Median	M ± SD	Median	M ± SD	M ± SD
		(Range)		(Range)		(Range)		
Uncomplicated idiopathic anterior uveitis	1	1231	62 ± 5,1	951	45,4 ± 5,2	257	15,4 ± 6,2	3,4 ± 1,0
N=32		(1127-1414)		(794-1091)		(216-327)		
				32				
Complicated by hypertension idiopathic anterior uveitis	2	1153	62 ± 7,5	836	44,3 ± 5,6	340	15,8 ± 5,7	3,2 ± 1,3
N=7		(1034-1206)		(820-936)		(252-374)		
Fuchs heterochromic uveitis	3	1893	62 ± 5,0	1059	50,0 ± 5,0	353	13,7 ± 4,0	4,3 ± 0,2
N=5		(1635-1893)		(1327-1509)		(308-395)		
Posner Schlossman syndrome	4	1248	66 ± 1,2	1053	53,2 ± 1,2	205	11,6 ± 2,1	4,7 ± 0,9
N=6		(1206-1354)		(936-1107)		(200-252)		
Control	5	1007	64,8 ± 5,8	645	43,1 ± 5,7	274	18,2 ± 5,4	2,5 ± 0,9
N=27		(772-1104)		(575-736)		(198-322)		
Level of Significant Differences		Р1-5=0,001		Р1-5=0,05	Р3-5=0,03	Р4-5= 0,006	Р1-5=0,08	Р1-5=0,003
		Р2-5=0,003		Р3-5=0,02	Р4-5=0,0001	Р3-4= 0,02	Р4-5= 0,01	Р2-5=0,04
		Р3-5=0,006		Р4-5=0,06	Р1-4=0,002			Р3-5=0,02
		Р4-5=0,002			Р2-4=0,009			Р4-5=0,006
								Р1-3=0,02
								Р1-4=0,002
**Note:** Median = median; Ql-Qup = lower-upper quartile (25%-75%); M = arithmetic mean; SD = standard deviation; N = number of patients								

The relative number of natural killers did not differ significantly from the norm, except for the group with Posner-Schlossman syndrome, in which this indicator was higher than the norm and there was a tendency to higher rates in comparison with the first (P1-4 = 0.07) and the second (P2-4 = 0.06) groups. The level of phagocytic neutrophils in the group of uveitis with intraocular hypertension was higher than normal by 25,3% (p<0,05).

**Table 3:** Level of natural killers and phagocytic neutrophils in patients with recurrent anterior uveitis, Fuchs and Posner-Schlossman syndrome during relapse

Nosological form	Group number	СD 16 (natural killers)		Phagocytic neutrophils	
		cells/ μL	%	cells/ μL	%
		Median	M ± SD	Median	M ± SD
		(Range)		(Range)	
Uncomplicated idiopathic anterior uveitis	1	194	10,4 ± 3,3	2600	73,8 ± 11,8
N=32		(155-256)		(2013-3423)	
Complicated by hypertension idiopathic anterior uveitis	2	220	12,0 ± 3,1	3488	84,6 ± 4,6
N=7		(180-272)		(2657-3952)	
Fuchs heterochromic uveitis	3	642	16,7 ± 5,7	1825	65,3 ± 20,2
N=5		(237-680)		(1825-7699)	
Posner Schlossman syndrome	4	156	8,0 ± 2,0	2257	77,2 ±10,2
N=6		(123-180)		(1909-5814)	
Control	5	196	12,4 ± 3,9	2926	67 ± 5,2
N=27		(102-233)		(2162-2954)	
Level of Significant Differences			Р3-5=0,02		Р2-5=0,0002
			Р2-3=0,04		
			Р3-4=0,02		
			Р1-3=0,04		
**Note:** Median = median; Ql-Qup = lower-upper quartile (25%-75%); M = arithmetic mean; SD = standard deviation; N = number of patients					

Analysis of changes in humoral immunity showed an increase in the absolute number of B-lymphocytes in groups 1-4. Groups with Fuchs uveitis and Posner-Schlossman syndrome showed highest values of B-lymphocytes (**Table 4**). The level of Ig A in groups 1-3 was above the norm by 32% (p<0,05), and in the Posner-Schlossman group it was above the norm by 63% (p<0,05). The level of Ig M in groups 1,3,4 was above the norm by 35% (p<0,05). The level of Ig G was reduced in the second group in comparison with the norm by 31% (p<0,05) (**Table 4**).

A weak direct correlation was found between the volumetric blood filling of the eye RQ and the level of Ig A in the blood serum of patients r=0.28 (p<0,05) and the level of Ig G r=0.27 (p<0,05).

Thus, features of humoral immunity were its high activity in idiopathic and Fuchs uveitis and Posner-Schlossman syndrome in terms of the number of B-lymphocytes, the level of Ig A and Ig M. A characteristic feature was a higher value of these indicators in Fuchs and Posner-Schlossman syndromes. IgG levels were deficient (below normal) in the idiopathic uveitis group with hypertension.

**Table 4:** Indicators of humoral immunity in patients with recurrent anterior uveitis, with Fuchs and Posner-Schlossman syndrome during relapse

Nosological form	Group number	СD 19 (В-lymphocytes)		Ig A		Ig M		Ig G
		cells/ μL	%	г/ л	г/ л	г/ л
		Median	M ± SD	M ± SD	M ± SD	M ± SD
		(Range)				
Uncomplicated idiopathic anterior uveitis	1	354				
N=32		(275-431)	16,5 ± 3,4	2,6 ± 0,7	1,2 ± 0,2	13,6 ± 1,9
Complicated by hypertension idiopathic anterior uveitis	2	360				
N=7		(260-374)	14 ± 4,4	2,5 ± 0,7	1,1 ± 0,3	9,8 ± 1,8
Fuchs heterochromic uveitis	3	514				
N=5		(450-514)	17 ± 1,7	2,5 ± 0,7	1,2 ± 0,4	11,7 ± 2,1
Posner Schlossman syndrome	4	371				
N=6		(360-389)	19,4 ± 0,5	3,1 ± 0,1	1,3 ± 0,08	12,5 ± 3,5
Control	5	236				
N=27		(178-278)	15,7 ± 3,4	1,9 ± 0,5	0,9 ± 0,2	14,4 ± 2,3
Level of Significant Differences		Р1-5=0,0001	Р4-5=0,02	Р1-5=0,0001	Р1-5=0,0001	Р2-5=0,0003
		Р2-5=0,03	Р1-4=0,07	Р2-5=0,04	Р2-5=0,03	Р1-2=0,0001
		Р3-5=0,005	Р2-4=0,03	Р3-5=0,03	Р4-5=0,00007	
		Р4-5=0,001	Р3-4=0,02	Р4-5=0,00001		
		Р1-3=0,03		Р2-4=0,06		
		Р2-3=0,02		Р3-4=0,001		
		Р3-4=0,03				
**Note:** Median = median; Ql-Qup = lower-upper quartile (25%-75%); M = arithmetic mean; SD = standard deviation; N = number of patients								

When analyzing the sensitivity of T-cells to retinal antigens and to antigens of the choroid, an increase in this indicator was revealed in all groups in comparison with the norm by 2,9-2,3 times (p<0,05), and in the group of patients with Posner Schlossman syndrome - 3,8-4 times (p<0.05) (**Table 5**). The sensitivity of T cells to lens antigens was higher than normal in groups of uveitis with intraocular hypertension and Posner Schlossman syndrome by 1,9 times (p<0.05) and higher than in groups of uncomplicated uveitis and with Fuchs uveitis by 50,7% (p<0.05) (**Table 5**). Thus, there was a high sensitivity to eye tissue antigens in patients with anterior uveitis, most pronounced in patients with Posner-Schlossman syndrome. The high sensitivity to the lens antigen in patients with intraocular hypertension and Posner Schlossman syndrome should be noted.

**Table 5:** Peripheral blood T-cell sensitivity to retinal (antigen of retina - aR), choroidal (antigen of choroid - aCh) and lens (antigen of lens - aL) antigens in patients with recurrent anterior uveitis, with Fuchs uveitis and Posner-Schlossman syndrome during the relapse

Nosological form	Group number	aR	aCh	aL
		%	%	%
		M ± SD	M ± SD	M ± SD
Uncomplicated idiopathic anterior uveitis	1	12,9 ± 4,6	11,9 ± 3,8	12,5 ± 5,1
N=32				
Complicated by hypertension idiopathic anterior uveitis	2	12,6 ± 5,1	12,0 ± 4,4	18,0 ± 7,3
N=7				
Fuchs heterochromic uveitis	3	10,7 ± 1,1	12,0 ± 0,8	12,6 ± 2,3
N=5				
Posner Schlossman syndrome	4	17,2 ± 6,2	21,2 ± 9,3	20 ± 8,3
N=6				
Control	5	4,5 ± 3,4	5,3 ± 4,6	10 ± 3,7
N=10				
Level of Significant Differences		Р1-5=0,0001	Р1-5=0,0001	Р2-5=0,004
		Р2-5=0,006	Р2-5=0,003	Р4-5=0,002
		Р3-5=0,009	Р3-5=0,003	Р1-2=0,02
		Р4-5=0,00001	Р4-5=0,002	Р3-4=0,04
		Р1-4=0,07	Р1-4=0,0001	
		Р3-4=0,08	Р2-4=0,01	
			Р3-4=0,02	
**Note:** M = arithmetic mean; SD = standard deviation; N = number of patients					

## Discussion

In the presented study, the incidence of idiopathic recurrent anterior uveitis complicated by intraocular hypertension was 9,9% among all cases of idiopathic recurrent anterior uveitis in one-time period. According to literature, this complicated form of uveitis occurs in 11,5%-46,1% of cases. Most often (up to 92% of cases), the anterior chamber angle was open [**12**,**27**,**28**]. All our patients also had an open anterior chamber angle. According to our data, intraocular hypertension occurred in 100% of cases with Posner-Schlossman syndrome and Fuchs heterochromic uveitis. Such a high incidence of hypertension is consistent with literature data [**12**]. The reported high frequency of elevated IOP indicated the importance of careful monitoring in uveitis.

**Fig. 1:**
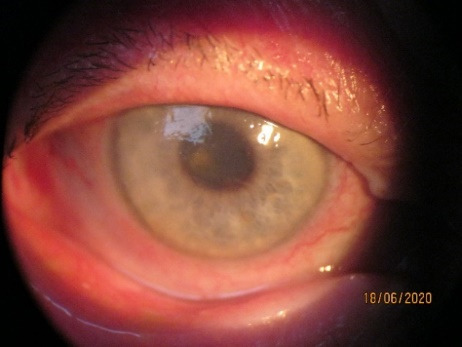
RE (OD)

**Fig. 2:**
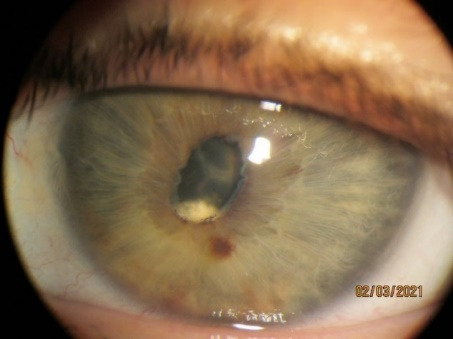
LE (OS)

To show some interesting cases from our practice, we presented Patient 1, of 35 years old. She had uveitis and uveal glaucoma due to rheumatoid arthritis and was treated with steroids (orally, eye drops, intramuscularly). OS (left eye) was operated for cataract, but the eye lost function due to a complete retinal detachment. IOP in the right eye had risen. IOP stabilized after the disappearance of uveitis. Visual acuity stabilized despite progressive lens opacity (0,5 nc). Patient 1 continued to be treated by a rheumatologist [**8**]. The figures below show her eyes on anterior segment photographs (**Fig. 1**,**2**).

**Fig. 3:**
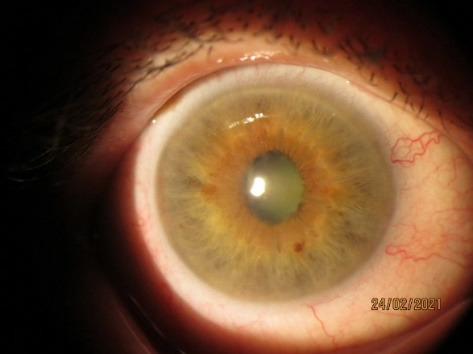
RE (OD), 24.02.2021

**Fig. 4:**
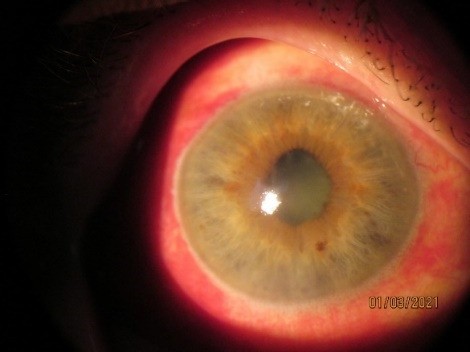
RE (OD), 01.03.2021

Patient 2, RM, 60 years old, was observed since 2015 for chronic uveitis in OU (recurrent OD, remission OS). The patient’s medical history showed an increase in IOP, for which she was treated with antibiotics, steroids, NSAIDs, and was observed in the Republic of Moldova and sometimes in Italy and Germany. After frequent relapses in 2017, pupillary fusion occurred in the OD, iris bombe with IOP of 36 mmHg and complicated cataract (**Fig. 3**,**4**). Laser iridotomy and anterior chamber injection of Bevacizumab were performed. OD calmed down, only pigmented precipitates remained. Currently, IOP is 20/ 20 mmHg. This case shows the possibility of development of uveitic glaucoma, in a patient with chronic uveitis [**8**].

As usually, uveitis associates inflammation, vascular hyperpermeability, sometimes with occlusion and eye ischemia because of inflammatory cytokines. Early diagnosis can prevent severe complications such as macular edema and new vessels formation. The study of hemodynamic features can improve our knowledge of the pathophysiology and natural course of the disease, and guide the decision-making of an ophthalmologist dealing with uveitis in the right direction [**29**].

Currently, due to the anatomical features of the vascular system of the eye, there is a small selection of non-invasive methods for studying eye-hemodynamics, which determine its functional characteristics such as Fluorescein angiography (FA) and optical coherence tomography (SD-OCT). They are the most common ways to detect complications in these patients [**30**,**31**]. The transition from SD-OCTA to SS-OCTA has made visualization of choroidal anatomy more accessible and improved image quality for quantitative analysis. Sometimes, because of media opacities, some difficulties in taking good photos in patients with uveitis appear [**32**]. At present, few studies using angio-OCT in uveitis exist. While conducting a quantitative analysis, it was revealed that there is a significantly greater deficit of choroidal blood flow than in other forms of uveitis in posterior uveitis [**33**].

In cases with proliferative process in the choroid, a significant decrease in the maximum and minimum blood flow velocities in the posterior short and long ciliary arteries was noted in comparison with the control group. This was possible by studying the state of regional hemodynamics using Doppler ultrasound of the eye vessels in chorioretinitis of various etiologies [**34**].

Using a non-invasive method of impedancemetry (rheoophthalmography), we determined the state of volumetric blood filling of the choroid in terms of RQ. The analysis revealed a correlation between the values of the RQ index and the period of the disease - remission or relapse. Different levels of blood supply to the eye are determined in patients with uncomplicated and complicated increased IOP anterior uveitis, which is characterized by its insufficiency during remission. In case of uveitis with intraocular hypertension, its insufficiency is persistently expressed as there is no difference between remission and relapse. In Fuchs and Posner-Schlossman syndromes, no significant changes were detected during the relapse period. All these reflect the different nature of pathophysiological processes and the participation of the vascular system in the inflammatory process with a heterogeneous clinical aspect.

In the last three decades, the state of T-cell subpopulations in peripheral blood in anterior uveitis has been widely studied. The results of these works are contradictory. Compared with the norm, no changes were detected in the total number of T cells and in the ratio of helper/ suppressor cells of peripheral blood T cells in idiopathic uveitis and Fuchs uveitis [**35**,**36**], and a decrease in the number was found by a decrease in the total number of T lymphocytes in half of the patients who also had a deficiency of T-helpers in uveitis [**37**].

For comparison, some data showing that the vast majority of HBV-infected patients, especially those with inflammatory forms of eye pathologies, and who have local and systemic disorders of the immune status, are presented. Characteristic and common for the majority of HBV-infected patients, regardless of the clinical form of the eye disease, were the following: weakening of T-cell immunity (decrease in the total number of T-lymphocytes, lack of T-helpers); imbalance of immunoregulatory subpopulations, mainly due to deficiency of CD4-, less often - CD8-cells), causing a decrease (in the vast majority of patients with keratitis and in 43% with uveitis) or an increase (in 26.8% with uveitis) of the index SD4/ SD8; a significant increase in the concentration of the CEC; serum dysimmunoglobulinemia (mainly IgG and/ or IgA, rarely IgM); increased systemic production of TNF-a while weakening the ability for systemic and local production of antiviral IFN-a - a key factor in antiviral immunity; deficiency of IgA in the SF with increased secretion of IgG.

Manifestations of immunopathology characteristic of uveitis associated with HBV infection, patients with CG of unknown etiology and distinguishing this group from patients not infected with hepatitis viruses and not suffering from CG were revealed. These include a deeper weakening of T-immunity, manifested by a decrease in the total number of T-cells, active T-lymphocytes and T-helper subpopulations (CD4); more frequent imbalance of immunoregulatory subpopulations towards a decrease or increase in the CD4/ CD8 index. More often than in other patients with uveitis, in the group of HBV-infected patients, elevated CEC concentrations were detected (in 16% and 33.7%, respectively; p<0.05) and also, in general, rare signs of immunopathology, such as the presence in the SC and/ or in the SF of antibodies to myelin (11.1%), collagen components (11.5%) and liver (17.5%). A deeper deficiency of IFN-f with activation of systemic and local production of the anti-inflammatory cytokine -TNF-a, which was observed in almost 90% of cases, was characteristic for HBV-infected patients, while in other etiology of the disease - half as often.

It is known that in experimental autoimmune uveitis, T-lymphocytes, especially CD4 + T-lymphocytes, play a central role in its immunopathogenic mechanisms. In humans, activated CD4+ T cells and the inflammatory cytokines produced by these activated T cells also play a central role in immunopathogenic mechanisms [**38**]. Such differences can probably be explained by the fact that idiopathic uveitis is a very diversified disorder, it is a diagnosis of exclusion when clinical, radiological and biological examinations are inconclusive, and ophthalmological examination is not pathognomonic for a particular disease. Some idiopathic uveitis may be autoimmune or undiagnosed infectious uveitis, may have genetic determinants, or be the result of environmental factors. Currently, an infectious agent is considered as an initiating or potentiating factor. One way in which microbial agents may play a role is through their adjuvant effect, for example in shifting the balance of immune responses that are normally controlled by inhibitory regulatory mechanisms towards mechanisms that predispose patients to develop one of these diseases [**4**,**23**,**39**,**40**]. Common to the Posner-Schlossman and Fuchs syndromes is elevated IOP, which can lead to secondary glaucoma. It was found that, in general, inflammation in both diseases was promoted by a strong Th1 response, and high levels of IFN-γ, IL-5, and IL-10 in Posner-Schlossman syndrome indicate a distinctly more acute inflammation [**41**-**43**]. In our study, the immunoregulatory index was higher in the group of patients with Posner-Schlossman syndrome and Fuchs uveitis than in patients with uncomplicated idiopathic uveitis. At the same time, the highest values of humoral immunity were higher in these groups. Also, the number of natural killers in the group with Posner-Schlossman syndrome was also increased. All these point to the active stimulation of the immune system in these syndromes, as well as to various mechanisms of the immune response in uveitis complicated by an increase in IOP. This is confirmed by literature data - studies of the local immune response: in the intraocular fluid of the anterior chamber in Fuchs uveitis, CD8+ T cells were higher than in idiopathic uveitis, and the number of CD4+ cells was higher in idiopathic anterior uveitis than in Fuchs uveitis. Various local mechanisms have been proposed to regulate the balance of T-cell/ cytokine-mediated inflammation in the anterior segment, which may underlie clinical differences, such as chronicity and steroid response in these diseases [**36**]. We have studied the sensitivity of T-cells to retinal antigens, choroid, and lens. High sensitivity to these eye tissue antigens was found in patients with anterior uveitis, most pronounced in patients with Posner-Schlossman syndrome. High sensitivity to the lens antigen should be noted in patients with intraocular hypertension and Posner Schlossman syndrome. According to literature data, in the experiment, immunization with retinal antigens activates antigen-specific retinal T cells. Over time, these activated T cells produce inflammatory cytokines, especially T helper (Th)1 cytokines, such as interferon (IFN)-γ and interleukin (IL)-2, which attract inflammatory cells such as B cells, macrophages, and retinal microglia, which can then damage retinal tissue. Thus, as intraocular inflammation develops, activated T cells and antigen-specific T cells are present in the retina [**42**-**45**]. As a result, we studied the sensitivity of peripheral blood T cells.

It should be noted that we revealed a weak correlation between the volumetric blood filling of the eye RQ and humoral immunity (Ig A and Ig G levels) in patients with anterior uveitis. 

## Conclusions

1) Volumetric blood supply to the eye according to the rheoophthalmographic indicator RQ during remission of uncomplicated and complicated by hypertension anterior uveitis was reduced by 32.4%-40.5%, respectively, compared with the norm. In uveitic patients without complications, in remission period, RQ was significantly higher by 28% (p<0.05) than in remission, and in the group of uveitis with increased IOP, no significant differences were noted between the periods of remission and relapse, which reflected the ischemic process in the relapse period. Volumetric blood filling in Fuchs and Posner-Schlossman syndromes in the acute period did not differ from the norm.

2) A feature of cellular immunity in the groups of uncomplicated and complicated by intraocular hypertension idiopathic uveitis, as well as with Fuchs and Posner-Schlossman syndromes, is a higher level of CD4 helper lymphocytes and a lower level of CD8 suppressor lymphocytes, which reflect higher values of the immunoregulatory index. The increase in the immunoregulatory index is most pronounced in Fuchs and Posner-Schlossman syndromes.

3) A feature of humoral immunity is its high activity in idiopathic uveitis, Fuchs and Posner-Schlossman syndromes in terms of the number of B-lymphocytes and the level of Ig A and Ig M.

4) Characteristic only for idiopathic uveitis, complicated by intraocular hypertension during the relapse period, was an increase in the level of phagocytic neutrophils and a deficiency in the level of Ig G in peripheral blood serum.

5) A high sensitivity to eye tissue antigens (retina, choroid, lens) was revealed in patients with anterior uveitis, most pronounced in patients with Posner-Schlossman syndrome. Higher sensitivity to the lens antigen was also observed in patients with uveitis with intraocular hypertension in comparison with its uncomplicated form.

**Conflict of Interest statement**

The authors report no conflicts of interest for this work.

**Informed Consent and Human and Animal Rights statement**

All participants signed a written informed consent.

**Authorization for the use of human subjects**

Ethical approval: The study protocol was approved by the Ethics Committee of the SI “The Filatov Institute of Eye Diseases and Tissue Therapy of the National Academy of Medical Sciences of Ukraine” (protocol № 2 of 2022), and was performed according to the principles of safety, ethical attitude and application of the rules of working with patients in accordance with the “Bioethical Regulations of the Declaration of Helsinki on the ethical regulation of medical research”, the Convention of the European Council on Human and Biomedical Rights and the relevant Laws of Ukraine.

**Acknowledgements**

None.

**Sources of Funding**

No funding was received for this research.

**Disclosures**

None. 
